# Editorial: An outlook on urobiome: advances in understanding the role of urobiome in urological health and disease and its potential in biotherapeutics

**DOI:** 10.3389/fruro.2024.1380340

**Published:** 2024-02-23

**Authors:** Harris Onywera, Ramadhani Chambuso, David J. Benjamin, Evann E. Hilt, Krystal Thomas-White

**Affiliations:** ^1^ Institute of Infectious Disease and Molecular Medicine, University of Cape Town, Cape Town, South Africa; ^2^ Division of Medical Microbiology, Department of Pathology, Faculty of Health Sciences, University of Cape Town, Cape Town, South Africa; ^3^ Research, Innovations, and Academics Unit, Tunacare Services Health Providers Limited, Nairobi, Kenya; ^4^ Centre for Research in Infectious Diseases, College of Graduate Studies and Research, Mount Kenya University, Thika, Kenya; ^5^ Department of Global Health and Population, Harvard T. Chan School of Public Health, Boston, MA, United States; ^6^ Hoag Family Cancer Institute, Newport Beach, CA, United States; ^7^ Department of Laboratory Medicine and Pathology, University of Minnesota Medical Center, Minneapolis, MN, United States; ^8^ Research and Development, Evvy, New York, NY, United States

**Keywords:** urobiome, uropathogen, urinary tract infection (UTI), urological, urogenital, health, disease

## Kicking it off: current outlook on the human urobiome

High-throughput, culture-independent technologies such as next-generation sequencing of the bacterial 16S ribosomal RNA (rRNA) gene, have become a phenomenal milestone in the exploration of the structure, function, and diversity of the microbiome of various body sites (1). Despite these recent strides, the bladder, urethra, and urinary microbiome (urobiome) remains relatively understudied; yet it may contribute to urological infections and diseases (2, 3), including urinary tract infections (UTIs), the most common outpatient infections (4). Available literature on urobiome exhibits heterogeneity in study methodologies, with a majority of studies having relatively small sample sizes and focusing on associations rather than causations. There are other fundamental questions that are yet to be examined. For instance, the origin, temporal stability, and individuality of the core urobiome, its dynamic and intricate interplay with the host immunity, urinary incontinence and various uropathologies, other factors (e.g., host genetics) impacting it, how its individual microbial communities (bacterial, virome, and mycobiome) biologically interact, and its correspondence with microbiome of other body sites such as the gut and penis, remain largely unexplored (2, 5). Therefore, a comprehensive understanding of the urobiome using more large-scale and well-designed follow-up studies with appropriate controls and standardized methodologies are warranted. This will allow for generalizability of the findings to the wider population and cross-study comparisons. Importantly, the study findings have potential applications in diagnostics, prognostics, and therapeutics. The attention of the present Research Topic includes urobiome in health and disease, novel methodologies for exploring urobiome, uropathogens in public health, and urobiome in translational science. This Editorial explores and synthesizes four articles – one brief research report (Baddoo et al.), two reviews (Brubaker et al. and Moreland et al.), and one original research (Maxwell et al.) – submitted, peer-reviewed, and published on this topic. These articles, presented below, underline the need to explore the frontiers of urobiome research and UTI diagnosis with standardized and improved toolkits to revolutionize urogenital health.

### Cataloging variation in 16S rRNA gene sequences of female urobiome: insights from short-read sequencing

Potential biases and taxonomic uncertainties in determining microbiome composition and diversity using various 16S rRNA hypervariable regions, sequencing technology, and bioinformatics pipelines have been underscored (6). Baddoo et al. sought to assess short-read sequencing accuracy of frequently used 16S rRNA hypervariable regions (V1-V3, V4, and V4-V6) in characterizing female urobiome. The choice of these three regions was based on a previous evaluation of the achievable taxonomic resolution for bacterial species in the female urinary tract. First, the investigators had 203 isolates from the bladder urine – collected via transurethral catheterization – that were plated on differential media. MALDI-TOF mass spectroscopy (MS) was used for rapid colony identification (genus prediction). Thereafter, DNA was extracted from 1,088 morphologically distinct colonies, 16S rRNA gene amplified, followed by full-length sequencing on a Sanger platform. This resulted in 831 high-quality sequences (average length: 1,250 bp), phylogenetically inferred as 69 distinct species and 33 genera. Baddoo et al. then developed a reference set of 399 unique full-length or near-full-length 16S rRNA sequences (representative of 160 distinct species from 73 genera) by combining their dataset with publicly available genomes from the female urinary tract. With this reference set, they assessed the taxonomic resolution achievable using each of the three hypervariable regions. These regions unveiled varied accuracies in genus and species-level classifications, with V1-V3 region having a more reliable taxonomic resolution than V4-V6 region or V4.

While Baddoo et al. did not explore the performance of the other 16S rRNA hypervariable regions and configurations, their work highlights that the choice of the 16S rRNA hypervariable region and configuration may present limitations to the characterization of the urobiome. Also, full-length or near-full-length 16S rRNA gene sequencing offers deeper and more accurate taxonomic assignments than short-read sequencing. Owing to the proposition that urobiome differs by sex (7) and present lack of consensus on the optimal region to use for urobiome studies, it may be necessary for future investigators to also catalogue the variation in 16S rRNA gene sequences of male urobiome bacteria. Any reference set developed can be used to augment existing databases, by expanding urobiome species diversity.

### Tarnished gold—the “standard” urine culture: challenging the traditional view of UTI diagnosis and treatment algorithm

Brubaker et al. reflected on the importance of reexamining and improving UTI diagnostic testing to improve treatment outcomes. They underscored that SUC – the “gold standard” for UTI diagnosis – relies on a simplistic and an inaccurate dichotomy that frequently underreports fastidious bacteria. SUC test overlooks the complexity of urobiome, and when used on its own, may cause poor management of UTIs. Inappropriate antibiotic use (e.g., overtreatment) can cause antimicrobial resistance (AMR) and grossly perturb eubiotic urobiome and microbiome of other body sites such as the gut. The review emphasizes the need to prioritize the development of novel, more accurate, and timely UTI tests that can probe the complexity of the urobiome, and incorporate both rigorous microbiological insights and objective biological host-response data for improved patient outcomes, while upholding antibiotic stewardship. To improve UTI treatment outcome, we must: i) challenge the prevailing perception that the current UTI care is “adequate”, ii) acknowledge the limitations of SUC test, and iii) replace SUC test by more sensitive advanced methods such as metaculturomics and metagenomics, which can frequently detect a rich collection of commensals, opportunistic bacteria, and classical uropathogens.

### A call to global action: comprehensive understanding of uropathogens should go beyond the “usual suspects”

The deliberations by Moreland et al. augment and echo the reflections by Brubaker et al. They focused on poorly understood, emerging and suspected uropathogens; emphasizing the rise of multidrug-resistant strains. While commonly accepted uropathogens like *Escherichia coli* are briefly discussed, the focus is directed towards underresearched species, saprophytes and other environmental pathogens (e.g., *Acinetobacter baumannii*), *Candida* spp. (e.g., *C. albicans*), genus *Staphylococcus*, and emerging and suspected uropathogens (e.g., *Enterococcus faecalis*). Emerging uropathogens have been underappreciated primarily because: i) many fail to or poorly grow under SUC conditions and ii) accurate identification of many species was challenging prior to the age of MALDI-TOF MS and subsequent technologies. Some bacteria such as members of the family *Aerococcaceae* and *Actinomyces*-like organisms (e.g., *Schaalia*) are beginning to be reassigned accurate taxonomic identities following the reassessment of their phylogenetic positioning and chemotaxonomic characteristics. The review criticizes Koch’s postulates and the traditional concept of commensals versus pathogens, calling for a more comprehensive understanding of microbial interactions and pathophysiology in the urinary tract. Recent investigations using advanced techniques such as metaculturomics and metagenomics are compelling experts to reassess the dogma that has traditionally considered anaerobes from orders *Eubacteriales* and *Bacteroidales* as apathogenic. The paper stimulates research studies to identify risk factors and improve antibiotic surveillance, particularly for species without Clinical and Laboratory Standards Institute (CLSI) standards. With the advancement of new molecular diagnostic techniques, there is a need to review and expand the current catalogue of uropathogens.

Here, we also propose that new perspectives should unravel the mechanistic interactions between the uropathogens and other organisms, such as high-risk human papillomavirus (HR-HPV) detected in the urinary system. HR-HPV has been isolated in voided urine and bladder cancer tissue (3, 8), yet its interplay with the urobiome-immune axis in bladder cancer remains relatively uncharted (3). Deficiency in HR-HPV-specific CD8+ T-cells has been associated with high-grade intraepithelial neoplasia. Both CD4+ and CD8+ T-cell responses have been associated with HR-HPV oncoprotein E6-related lesion regression, while oncoprotein E7-regression is attributed solely to CD4+ T-cell responses (9, 10). The bladder’s immune system seems to be balancing the need to respond promptly to microbial challenge with the need to rapidly curtail inflammatory responses, as the structural integrity of the epithelial barrier is disrupted during prolonged immune responses (11). Bladder epithelial cells are known to alert the immune system during infection and directly mediate microbial clearance by secreting antimicrobial compounds into the urine and by expelling invaders back into the bladder lumen, reducing intracellular load (10, 11). Below are the key gaps in understanding the relationship between HR-HPV infection and changes in the immuno-urobiome during bladder carcinogenesis and its therapy:

- The causal relationship between HR-HPV infection and alterations in the immuno-urobiome and bladder carcinogenesis is not fully understood (10).
- The mechanisms by which the urobiome-immune interaction may influence the efficacy of HPV vaccination in preventing bladder cancer are yet to be elucidated (8, 9).
- There is a lack of clarity on how immunomodulatory therapies could be tailored based on the interplay between HR-HPV and the urobiome-immune axis in order to improve the outcomes in bladder cancer patients (3).

### Patient perspectives on recurrent UTI: biopsychosocial burden and healthcare disillusionment

Maxwell et al. utilized a large-scale, online, cross-sectional qualitative research survey (conducted between July 2019 and June 2021) to investigate the experiences of 1,983 international women (aged 18-99 years) with self-reported rUTI. Two primary themes were adduced: i) the patient burden of rUTI and (ii) healthcare disillusionment. The former entailed a complex biopsychosocial impact of the illness and encompassed four subordinate themes: *facing ongoing uncertainty of living with rUTI*; *symptom severity*; *intimate relationship distress*; and *perceived stigma*. Health disillusionment, which is currently unexplored in the literature, involved patient dissatisfaction with healthcare received. It encompassed three subordinate themes: *concerns about AMR due to frequent antibiotic use*; *diagnostic inconsistency and fragmented treatment pathways*; and *devalued patient perspectives by healthcare provider interactions*. The study calls for effective patient communication, standardized exploration of patient perspective, improved patient-centered care, shared decision-making, and a holistic rUTI management approach.

## Concluding remarks

**Figure 1:**
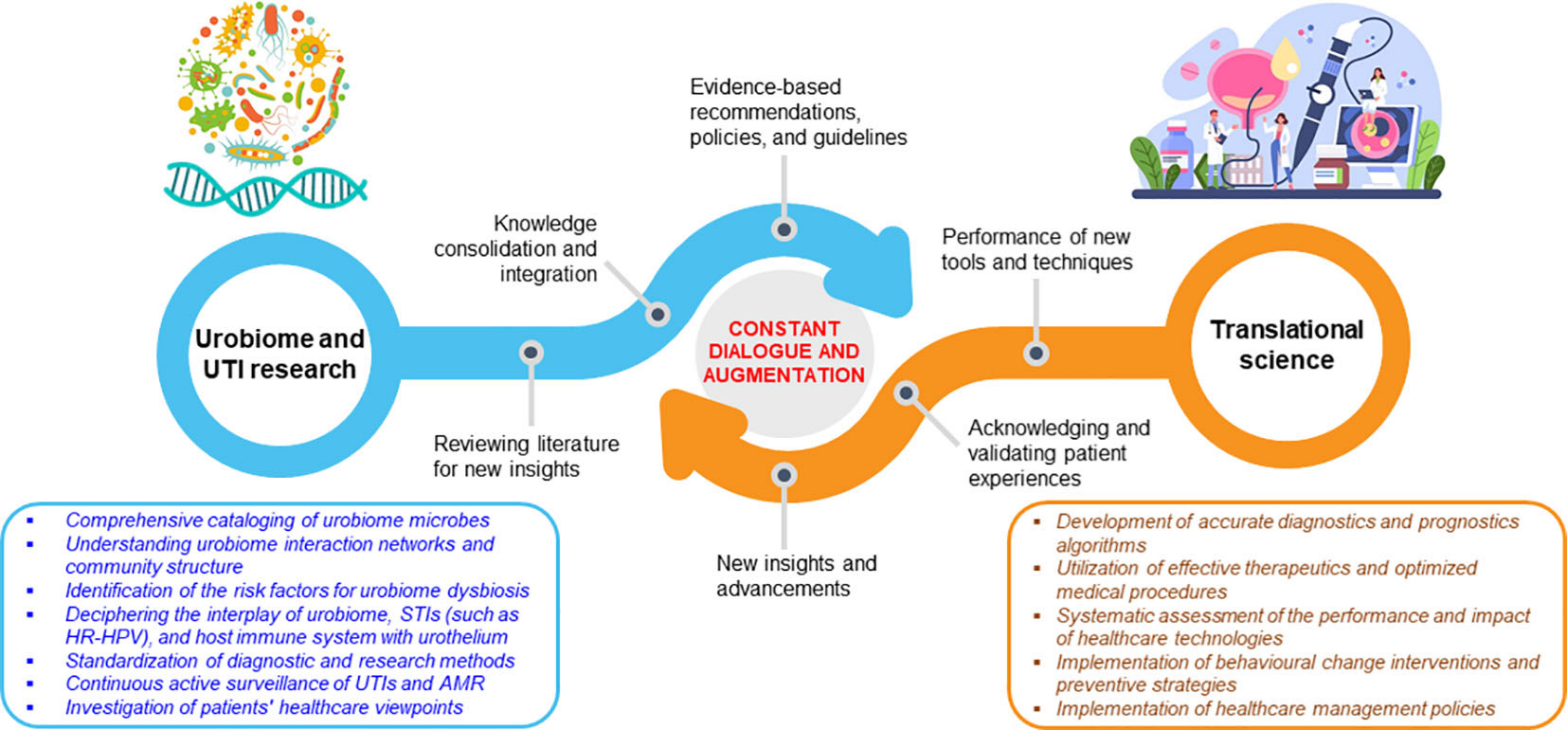
Continuous communication and augmentation between urobiome research and its translation. The dynamic exchange is guided by a tunable, evidence-dependent feedback system.

The articles discussed in this Editorial underscore the urgent need for consensus on optimal strategies for embarking on new frontiers of urobiome research (12). These include improved methodologies for characterizing the entire urobiome and complementary and/or alternative diagnostics tests that account for other uropathogens. Also, there is a need to develop actionable frameworks for management of patients with urologic infections, diseases, and disorders. The articles *en masse* highlight the need for a constant mutual interaction and augmentation between urobiome research and translational science (**Figure 1**). The above research and translation interplay, which is more complex than what is presently portrayed, can be leveraged to frame future urobiome studies and craft effective interventions to improve urogenital health.

## Author contributions

HO: Conceptualization, Visualization, Writing – original draft, Writing – review & editing. RC: Writing – review & editing. DB: Writing – review & editing. EH: Writing – review & editing. KT-W: Writing – review & editing.
